# The mitotic regulator polo‐like kinase 1 as a potential therapeutic target for c‐Myc‐overexpressing canine osteosarcomas

**DOI:** 10.1111/vco.12854

**Published:** 2022-08-23

**Authors:** Cecilia Gola, Luca Licenziato, Paolo Accornero, Selina Iussich, Emanuela Morello, Paolo Buracco, Paola Modesto, Luca Aresu, Raffaella De Maria

**Affiliations:** ^1^ Department of Veterinary Sciences University of Turin Grugliasco TO Italy; ^2^ SC Diagnostica Specialistica Istituto Zooprofilattico Sperimentale del Piemonte Liguria e Valle d'Aosta Turin TO Italy

**Keywords:** canine osteosarcoma, cell cycle, c‐Myc, polo‐like kinase 1, target therapy

## Abstract

Osteosarcoma is the most common primary malignant bone tumour in dogs, characterized by a locally aggressive and highly metastatic behaviour. Despite the current standards of care, most dogs succumb to the disease, indicating the need for novel treatment strategies. Polo‐like kinase 1 (PLK1) is dysregulated in a variety of human cancer types, including osteosarcoma, and induces c‐Myc accumulation. The crosstalk between the two molecules coordinates cell proliferation, differentiation, self‐renewal and apoptosis. Therefore, PLK1 has recently emerged as a potential therapeutic target, mainly in tumours overexpressing c‐Myc. BI 2536 is a selective PLK1 inhibitor promoting mitotic arrest and apoptosis in a variety of cancer cells. This research aimed at evaluating PLK1 and c‐Myc protein expression in 53 appendicular canine osteosarcoma (cOSA) samples and the in vitro effects of BI 2536 on a c‐Myc and PLK1‐overexpressing cOSA cell line (D17). PLK1 and c‐Myc expression in cOSA samples showed no correlation with clinicopathological data. However, c‐Myc overexpression was associated with a significantly reduced overall survival (*p* = .003). Western Blot and RT‐qPCR assays revealed that D17 expressed high protein and transcript levels of both *PLK1* and *MYC*. When treated with BI 2536 (range 2.5–15 nM) for 24 h, D17 showed a substantial decrease in cell growth, inducing apoptosis and G_2_/M cell cycle arrest. Interestingly, under BI 2536 treatment, D17 showed decreased c‐Myc protein levels. Consistent with human OSA, these preliminary data outline the prognostic value of c‐Myc expression in cOSA and highlight the potential role of PLK1 as an antiproliferative therapeutic target for tumours overexpressing c‐Myc.

## 1 INTRODUCTION

Osteosarcoma (OSA) represents the most common primary bone tumour in dogs^1^, ^2^, ^3^ and is characterized by a locally aggressive and highly metastatic behaviour.^1^ Despite the current standard of care, most dogs succumb to the disease within a year from the diagnosis.^1^ In addition, canine OSA (cOSA) shares several clinical, histopathological and molecular features with the human counterpart, including aberrant expression and mutations of driver genes,^2^, ^3^ hence representing an excellent model in comparative oncology.^1^ Polo‐like kinase 1 (PLK1) is a serine/threonine kinase^4^ playing a crucial role in cell cycle regulation and mitotic process^5^, ^6^, ^7^ by acting on chromosome segregation, spindle assembly and cytokinesis.^8^ Studies have demonstrated that PLK1 is usually overexpressed in a variety of cancers in human, including OSA (hOSA), and is broadly associated with a poor prognosis and disease progression.^9^, ^10^, ^11^ Conversely, its role in dogs remains unclear.

c‐Myc is a vital transcriptional regulator involved in cell cycle control, apoptosis, and protein synthesis. Additionally, *MYC* is one of the most commonly activated oncogenes in human and canine tumours being associated with tumourigenesis and sustained tumour growth.^12^, ^13^, ^14^ In hOSA, c‐Myc is frequently overexpressed and correlated with the development of metastases and a poor prognosis.^12^, ^15^, ^16^, ^17^ Although aberrant activation of c‐Myc pathway genes has been previously reported,^3^, ^13^ only recently MYC activation was correlated with a short disease‐free interval in cOSA.^14^

It is within this context that functional studies demonstrated that PLK1/Fbw7/c‐Myc axis creates a positive auto‐regulatory signal, sustaining the mutual increased expression of these genes.^18^, ^19^ In particular, PLK1 plays a key role in c‐Myc protein stabilization and accumulation in the cytoplasm, allowing its migration into the nucleus where it promotes G_2_/M transition and acting as a transcriptional factor.

These findings underline the importance of PLK1 inhibitors as promising selective therapies against c‐Myc‐overexpressing canine tumours, as previously demonstrated by the administration of Volasertib^20^, ^21^ in a number of human cancer subtypes.

In a recent RNA‐seq study, both *PLK1* and *MYC* oncogenes were found overexpressed in two well‐established cOSA cell lines, suggesting a potential implication of this signalling axis in this tumour.^3^ BI 2536, was the first selective PLK1 inhibitor able to promote mitotic arrest and apoptosis in MG‐63 human OSA cell line^22^ and xenografts models.^23^ Although Volasertib was developed from BI2536 given the more favourable pharmacokinetic properties,^20^ both molecules are potent PLK1 inhibitors with superimposable in vitro effects, including inhibition of c‐Myc expression and consequent cell death.^18^ These findings prompted us to investigate the role of PLK1 and c‐Myc in cOSA and to evaluate the in vitro biological effects of BI 2536 treatment^24^ on a PLK1 and c‐Myc‐overexpressing cOSA cell line.

## 2 MATERIALS AND METHODS

### 2.1 Sample collection and clinical data

Fifty‐three canine appendicular OSA samples were routinely collected at the Veterinary Teaching Hospital, Department of Veterinary Sciences (University of Turin), upon written consent from dog owners. All dogs were surgically treated with limb amputation or limb‐sparing techniques and received adjuvant chemotherapy (doxorubicin, cisplatin, or carboplatin as single agents or combinations). Thoracic radiographs or computed tomography (CT) evaluation was performed to exclude distant metastases prior to surgery. Follow‐up consisted of clinical evaluation and thoracic radiographs performed every 3 months during the first year and then every 6 months for a minimum of 2 years.

### 2.2 Histological diagnosis and immunohistochemistry

Tissues were placed in an EDTA‐based decalcification solution (Bio‐Optica, Milano, IT) until sufficient demineralization, before processing for histopathology. Formalin‐fixed, paraffin‐embedded (FFPE) tumour samples were stained with haematoxylin–eosin (HE) for diagnosis. The histological classification was performed according to the World Health Organization (WHO) guidelines^25^ and the grading was evaluated using the Loukopoulos and Robinson grading system^26^ by three independent pathologists.

Immunohistochemistry (IHC) was performed on 4 μm thick paraffin sections. After endogenous peroxidase activity blocking with 0.3% H_2_O_2_, the sections were exposed to heat‐induced antigen retrieval using citrate buffer at 98°C, pH 6 for 30 min and then incubated with anti‐PLK1 (PA5‐95265, Invitrogen, Waltham, MA, diluted 1:150) and anti‐c‐Myc antibodies (sc‐40, Santa Cruz Biotechnology, Dallas, TX, diluted 1:100) for 2 h at room temperature. Vectastain Elite ABC kit and ImmPACT DAB from Vector Laboratories Inc. (Burlingame, CA) were used for detection. All antibodies were validated for cross‐reactivity with canine positive controls^27^ (Figure S1).

Immunolabelled slides were randomized and masked for blinded examination, which was performed by two independent pathologists. Immunohistochemical evaluation of PLK1 and c‐Myc expression was performed using previously reported scoring systems and detailed in Table S1.^28^, ^29^

### 2.3 Cell line selection and culture conditions

Four primary cOSA cell lines were included in this study. Penny and Wall cell lines were previously established and validated by Maniscalco et al.,^30^ while the D17 (Cat.# ATCC CCL‐183) and D22 (Cat.# ATCC CRL‐6250) were obtained from American Type Culture Collection. These were cultured in Dulbecco's modified Eagle's medium (DMEM; D17 and D22) and Iscove's standard medium (Penny and Wall), supplemented with 10% foetal bovine serum (FBS), 1% glutamine, 100 μg/ml penicillin, and 100 μg/ml streptomycin. Cells were cultured at 37°C in a humidified atmosphere of 5% CO_2_. Human breast cancer cell line T47D (Cat.# ATCC HTB‐133) and hOSA cell line MG‐63 (Cat.# ATCC CRL‐1427), as well as a previously established osteoblast cell line (OSB),^31^ were used as controls.

### 2.4 Morphological changes, viability, and apoptotic assays after BI2536 treatment

The D17 cell line, known to overexpress PLK‐1 and c‐Myc proteins, was selected for inhibition experiments using BI 2536 (Boehringer Ingelheim, Ingelheim, Germany).^32^ A 10 mM stock solution of BI 2536 was prepared by resuspending the compound in dimethyl sulfoxide (DMSO). D17 cells treated with DMSO were used as control. First, 3 × 10^5^ cells/well were seeded in six wells cell culture plates and were then treated with BI 2536 at 2.5, 5, 7.5, and 15 nM for 12 and 24 h. Morphological changes were evaluated with a Leica AF6000 LX (Leica Microsystems, Wetzlar, Germany) microscope equipped with a Leica DFC350FX digital camera controlled by the LAS AF software (Leica Microsystems). Based on morphological effect, viability assay was performed using CellTiter 96 AQueous One Solution Cell Proliferation Assay (Promega, Madison, WI) on 1 × 10^6^ cells/well at 2.5, 5, 7.5, and 15 nM for 12 and 24 h. Similarly, caspase activity for apoptosis detection was evaluated using Caspase‐Glo 3/7 Assay System (Promega, Madison, WI) according to the datasheet after 12 and 24 h of treatment at 2.5, 5, and 7.5 nM. The untreated D17 cell line was used as control while the medium was used as blank to subtract background signal. All experiments were performed in triplicate and repeated three times.

### 2.5 PLK1 and c‐Myc expression in cOSA cell lines

The expression of PLK‐1 and c‐MYC in treated or untreated cells was evaluated by Western Blot (WB) and RT‐qPCR. Proteins from all the cell lines were extracted in lysis buffer (1% Triton X‐100, 10% glycerol, 50 mM Tris, 150 mM sodium chloride, 2 mM EDTA, pH 8.0 and 2 mM magnesium chloride) containing protease inhibitor cocktail (Sigma Aldrich, St Louis, MO). Twenty micrograms of total protein from all previous cell lines were separated by SDS‐PAGE (10% or 15%) and transferred onto a nitrocellulose membrane (Thermo Fisher Scientific, Waltham, MA). After washing, membranes were incubated in TBS/BSA 10% (bovine serum albumin) at room temperature for 1 h and incubated overnight at 4°C with PLK1 (PA5‐95265, Invitrogen, Waltham, MA, diluted 1:1000) and c‐Myc antibodies (5605T, Cell Signaling Technology, Danvers, MA, diluted 1:1000). β‐tubulin was used as an internal control (T5201, Sigma‐Aldrich, St Louis, MO, diluted 1:10 000). After incubation with horseradish peroxidase (HRP)‐linked secondary antibody diluted 1:15 000 in TBS‐Tween, membranes were washed 6 times in TBS‐Tween and incubated with Clarity Western ECL Substrate (Biorad Laboratories, Hercules, CA). The proteins were visualized by briefly exposing the membrane to an autoradiographic CL‐XPosure Film (Thermo Fisher Scientific, Waltham, MA). WB results were then acquired with an Epson scanner. T47D and MG‐63 cell lines, previously assessed expressing c‐Myc and PLK1, were used as positive controls.^22^, ^33^

Total RNA was isolated from all cell lines by using QIAzol Lysis reagent (Qiagen, Hilden, Germany). QuantiTect Reverse Transcription kit (Qiagen, Hilden, Germany) was used to retro‐transcribe 1 μg of total RNA into cDNA. RT‐qPCR was performed by using the IQ SYBR Green Supermix (BioRad Laboratories, Hercules, CA) and the IQ5 detection system (BioRad Laboratories, Hercules, CA). Primer sequences to determine *PLK1* and *MYC* transcripts were designed using Primer Express v. 1.5 software and are listed in Table S2. *GAPDH* showed stable expression levels under all experimental conditions and was selected as housekeeping gene. Gene expression was calculated using the formula 2‐ΔΔCt (fold increase), where ΔΔCt = ΔCt (sample) − ΔCt (control) and ΔCt was calculated by subtracting the Ct of the target genes from the Ct of the housekeeping gene. RT‐qPCR was performed in both technical and experimental triplicates.

### 2.6 Cell cycle analysis by FACS

The biological effect of BI 2536 on the cell cycle was evaluated by 7‐Aminoactinomycin D (7AAD) staining and FACS analysis. Briefly, D17 cells were exposed to 2.5, 5, 7.5, and 15 nM of BI 2536 for 16 h, detached with trypsin–EDTA, washed with PBS, fixed for 1 h at 4°C with 50% ice‐cold ethanol added drop‐by‐drop with continuous vortexing. Samples were then spun at 500 *g* for 7 min, resuspended in 1 ml of PBS with 25 μg/ml 7AAD and stained overnight at 4°C. The samples were analysed using a Cytoflex (Beckman Coulter) equipped with a 488 nm (Blue) excitation laser and a 690/50 nm (Red) emission filter. For each sample, 25.000–50.000 events were analysed, and each experiment was repeated 3 or more times. The percentages of cells in the different phases of their cycle were calculated using the Flowing Software version 2.5.1 (https://bioscience.fi/services/cell-imaging/flowing-software/).

### 2.7 Statistical analysis

Correlations of PLK1 and c‐Myc expression with clinical and histopathological data, as well as the mutual correlation of these factors, were analysed by Fischer's exact test. According to previously reported scoring systems, immunoreactivity for PLK‐1 and c‐Myc was classified in two categories for the statistical analyses. Additionally, Kaplan‐Meyer analyses were performed to examine the correlations of all variables with the time elapsed between surgery and the detection of metastases and/or local recurrence (disease‐free interval; DFI) and the time elapsed between surgery and death (overall survival; OS) using the log‐rank test, using R statistical software (R Core Team, 2018). Dogs who died for unrelated causes or were lost during the follow‐up were censored.

Data from viability and apoptotic assays, gene expression and cell cycle analyses were analysed by two‐way ANOVA to investigate the effect of BI2536 treatment. Data were analysed with GraphPad Prism (version 8.0.0, GraphPad Software). A *p* value of less than .05 was considered statistically significant.

## 3 RESULTS

### 3.1 Clinicopathological data

**FIGURE 1:**
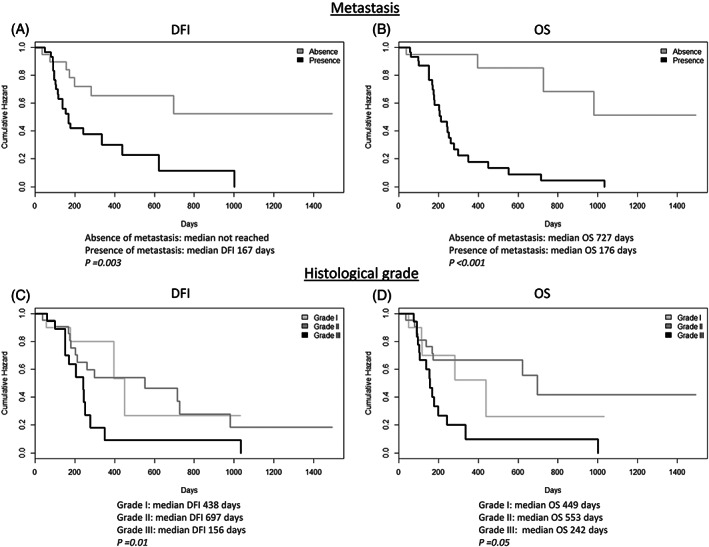
Kaplan–Meier curve. (A) Disease‐free interval (DFI) and (B) overall survival (OS), in dogs developing metastases compared to those without metastases; (C) DFI and (D) OS, in dogs with I, II, III grade osteosarcomas

This retrospective study included 53 dogs with appendicular OSA. The clinicopathological characteristics and follow‐up data are provided in Table 1. Both dogs bearing grade III OSAs and those developing lung metastases showed a significantly shorter DFI and OS than dogs with lower grade OSAs (grade I and II) and dogs without lung metastasis, respectively (Figure 1). The development of metastases was observed in 79% of dogs affected by grade III OSA, while reached 50% in OSAs with a lower histological grade (grades I and II).

**TABLE 1:** Clinicopathological characteristics of the dogs included in the study

Age (years)	Mean	7.5
	Median	8
	Range	2–13
Gender, *n* (%)	Female	24 (45.3)
	Male	29 (54.7)
Breed, *n* (%)	Crossbreed	13 (24.5)
	Boxer	6 (11.3)
	Rottweiler	5 (9.4)
	German Shepherd	5 (9.4)
	Great Dane	4 (7.6)
	Others breeds	20 (37.8)
Weight (kg)	Mean	39.9
	Median	38
	Range	7.5–71
Localisation, *n* (%)		
Forelimb of which	Total	33 (62.3)
	Proximal humerus	12 (22.6)
	Distal radius	10 (18.8)
Hindlimb of which	Total	20 (37.7)
	Distal tibia	10 (18.8)
	Distal femur	4 (7.6)
	Proximal tibia	1 (1.9)
Surgical treatment, *n* (%)	Amputation	47 (88.7)
	Limb sparing	6 (11.3)
Follow‐up (days)	Mean	214
DFI	Median	178
	Range	34–1493
	Mean	302
OS	Median	203
	Range	36–1493
Histological type, *n* (%)	Osteoblastic OSA	33 (62.3)
	Chondroblastic OSA	8 (15.2)
	Fibroblastic OSA	6 (11.3)
	Giant cell type OSA	2 (3.7)
	Poorly differentiated OSA	2 (3.7)
	Mixed OSA	1 (1.9)
	Telangiectatic OSA	1 (1.9)
Grading, *n* (%)	I Grade	11 (20.8)
	II Grade	22 (41.5)
	III Grade	20 (37.7)

### 3.2 c‐Myc is a negative prognostic marker in cOSA

**FIGURE 2:**
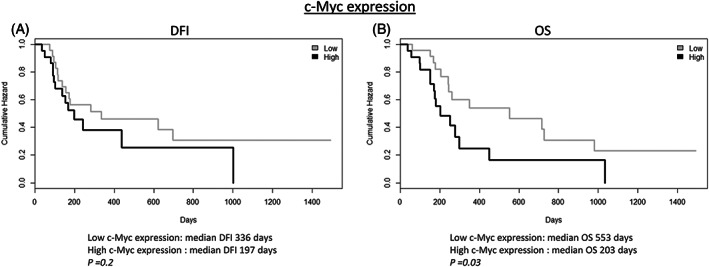
Kaplan–Meier curve. (A) DFI and (B) in dogs bearing high c‐Myc‐expressing osteosarcomas compared to low‐c‐Myc‐expressing osteosarcomas

**FIGURE 3:**
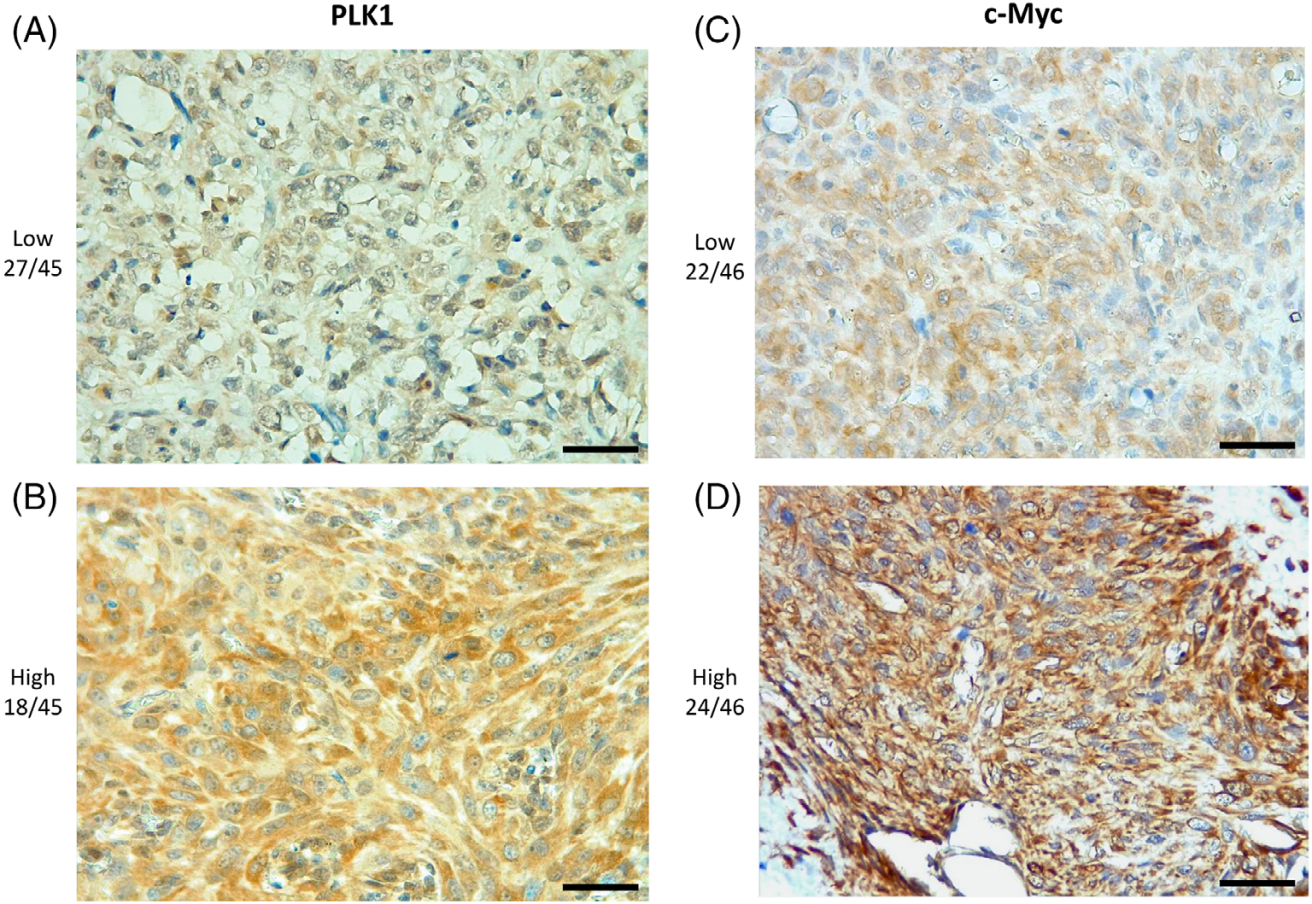
Immunohistochemistry. (A) Low and (B) high PLK1 expression in canine osteosarcoma samples; (C) Low and (D) high c‐Myc expression in canine osteosarcoma samples (scale bar = 50 μm)

A high c‐Myc expression by immunohistochemistry was correlated with a significantly shorter OS when compared to samples with a low c‐Myc expression (Figure 2). No other significant correlations with clinicopathological findings were found for both PLK1 and c‐Myc. A summary of IHC results of PLK1 and c‐Myc is provided in Table 2, and representative images are shown in Figure 3.

**TABLE 2:** Immunohistochemical scoring of PLK1 and c‐Myc

Marker	Positivity		Immunoreactivity		Total
	Score	*n* (%)	Score	*n* (%)	
PLK1	0	1 (2.1)	Low	29 (61.7)	47^a^
	1	1 (2.1)			
	2	5 (10.65)			
	3	7 (14.9)			
	4	5 (10.65)			
	6	10 (21.3)			
	8	11 (23.4)	High	18 (38.3)	
	12	7 (14.9)			
c‐Myc	0	3 (6.25)	Low	24 (50)	48^a^
	1	8 (16.65)			
	2	13 (27.1)			
	3	18 (37.5)	High	24 (50)	
	4	6 (12.5)			

a Immunohistochemical staining was not assessable in the remaining samples.

### 3.3 PLK1 and c‐Myc are broadly expressed in D17 and D22 cell lines

**FIGURE 4:**
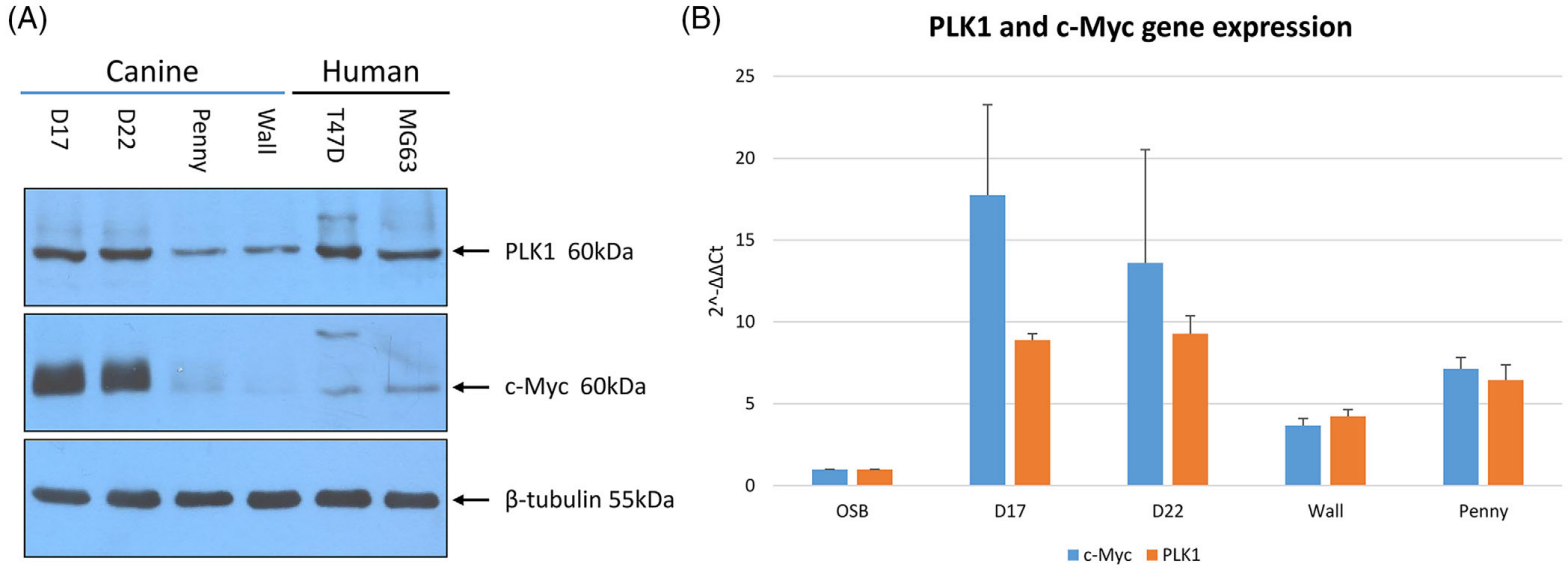
(A) Western Blot analysis of PLK1 and c‐Myc protein expression in untreated canine osteosarcoma cell lines (D17, D22, Penny and Wall) and human osteosarcoma and breast cancer cell lines (MG‐63 and TD47D, respectively). β‐tubulin was used as housekeeping gene. (B) Quantitative RT‐qPCR. Fold increase of both PLK1 and c‐Myc transcripts in D17, D22, Penny and Wall cell lines, compared to a canine osteoblast cell line. The error bars indicate the standard deviation of experimental triplicates

All cOSA cell lines, although at different levels, showed both PLK1 and c‐Myc expression. Overall, PLK1 and c‐Myc protein expression resulted higher in the D17 and D22 cell lines when compared to the Penny and Wall cell lines as well as to human MG‐63 and T47D cell lines (Figure 4). Quantitative PCR results confirmed these findings. Indeed, all cOSA cell lines expressed higher *MYC* and *PLK1* transcripts than canine osteoblasts, whereas D17 cells showed a greater amount of *PLK1* and *MYC* transcripts when compared to other cell lines (Figure 4).

### 3.4 BI 2536 induces G_2_/M cell‐cycle arrest and apoptosis in the D17 cell line

**FIGURE 5:**
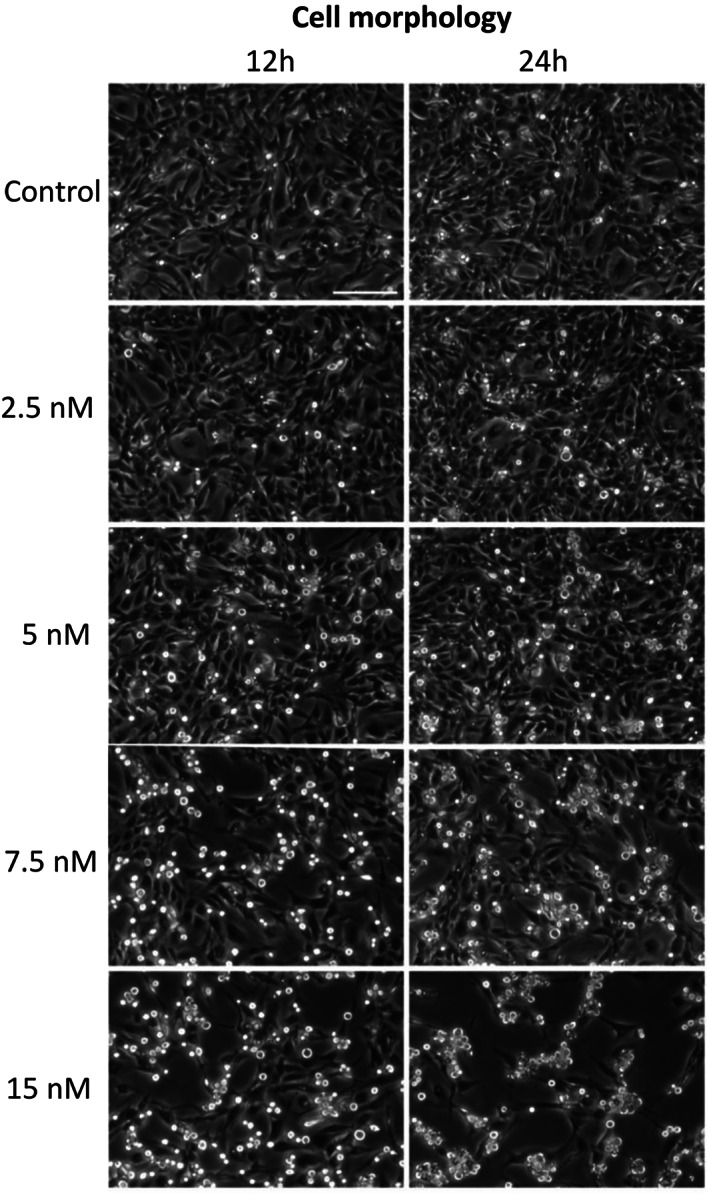
Cell morphology changes of the D17 cell line under BI 2536 treatment at different concentrations or untreated, for 12 and 24 h

**FIGURE 6:**
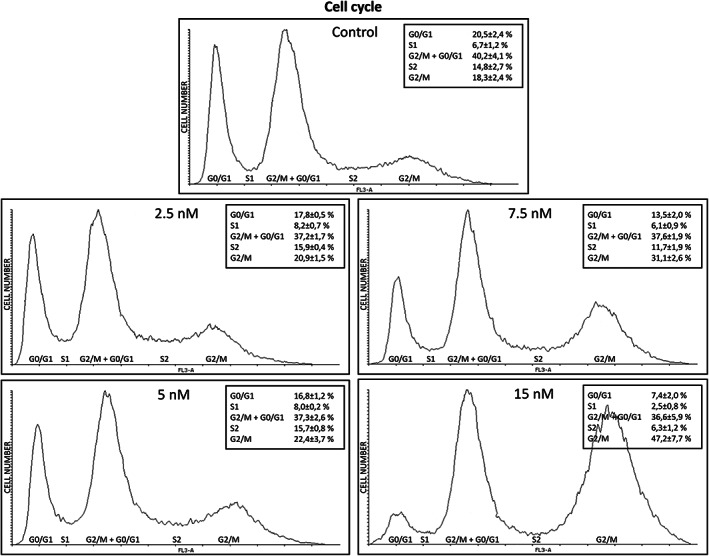
Cell cycle analysis of the D17 cell line under BI 2536 treatment at different concentrations or untreated, for 16 h

According to the aforementioned results, the D17 cell line was selected to evaluate the in vitro effects of BI 2536 on cell morphology, viability and apoptosis. As shown in Figure 5, BI 2536 treatment resulted in a significant change in cell morphology within 24 h characterized by an increased number of rounded‐up and floating cells. This phenomenon was more evident with higher BI 2536 concentrations and longer incubation times (Video S1). FACS analysis was performed to further confirm this finding and examine the impact of BI 2536 treatment on the cell cycle. Cell cycle analysis displayed a decrease of the cell population in the G_0_/G_1_ phase from 20% ± 2.4 in untreated cells to 7.4 ± 2% in cells treated with 15 nM BI 2536 for 16 h (*p* < .0001). Furthermore, a peak consisting of 37.8% ± 2.4 of cells and corresponding to the G_2_/M phase was observed in both untreated cells and under PLK1 inhibition. A third peak increasing from 18.3 ± 2.4% in control cells up to 47.2 ± 7.7% in cells under 15 nM treatment was also identified (*p* < .0001) (Figure 6).

**FIGURE 7:**
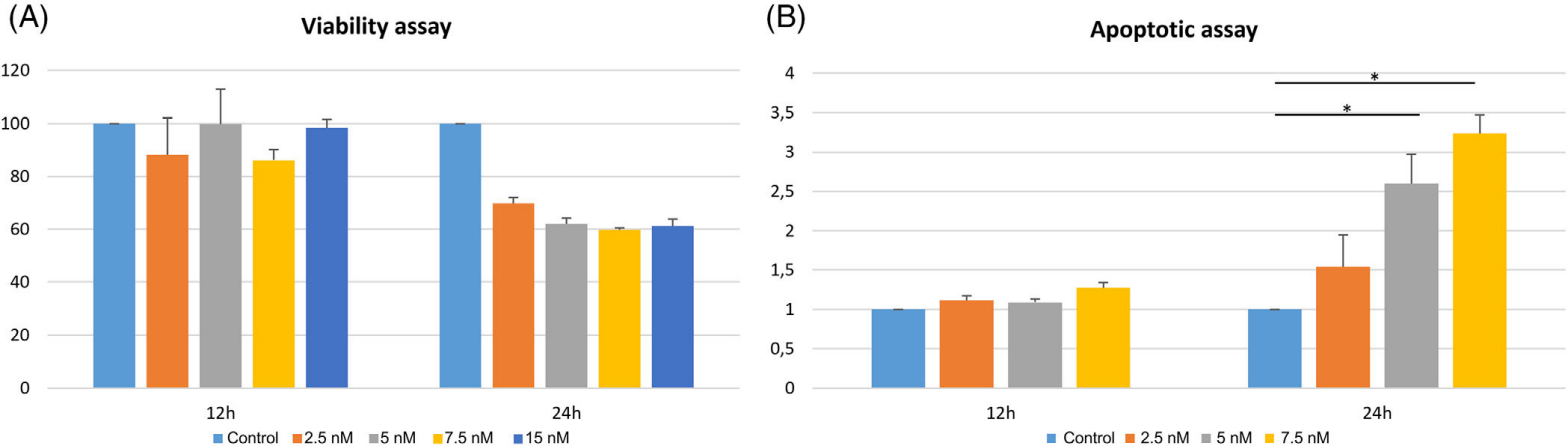
(A) Viability assay. Percentage of viable cells in the D17 cell line treated with BI 2536 at different exposure times and different concentrations, or untreated. (B) Apoptotic assay. Relative quantification of apoptosis in the D17 cell line treated with BI 2536 at different exposure times and different concentrations, or untreated (*p = .001). Data were normalized using the D17 untreated cell line in both assays; the error bars indicate the standard deviation of experimental triplicates

Cell viability and apoptosis assays were then performed. As shown in Figure 7 after 24 h of BI 2536 treatment in D17 we found a concentration‐dependent reduction of cell viability up to 61.2% when compared to untreated cells. Concurrently, a significant concentration‐dependent increase in apoptosis after 24 h treatment at 5 and 7.5 nM in D17 cells treated with BI 2536 was found (*p* < .0001).

### 3.5 PLK‐1 inhibition in vitro reduces c‐Myc protein expression

**FIGURE 8:**
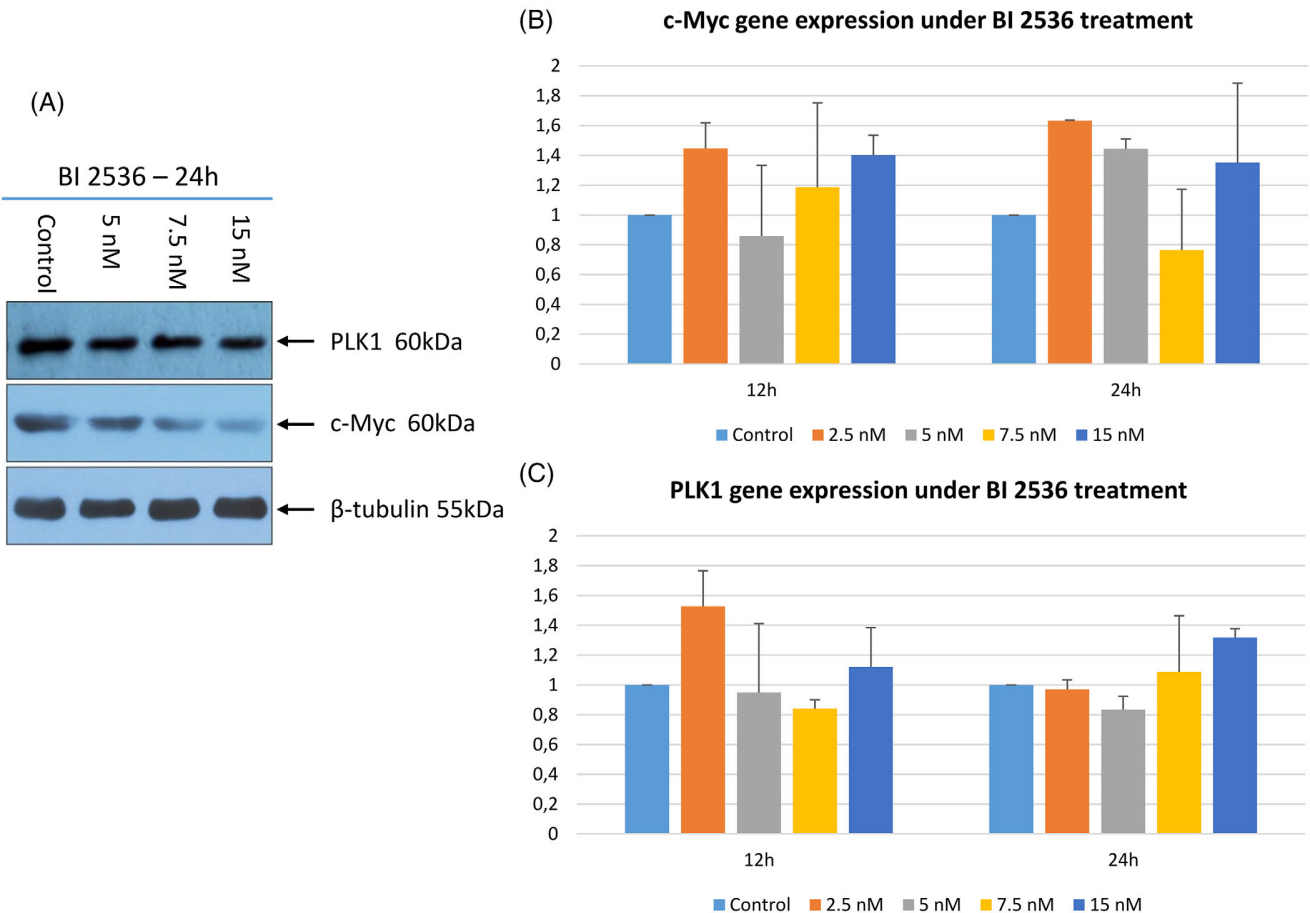
(A) Western Blot analysis of PLK1 and c‐Myc protein expression in the D17 cell line treated with BI 2536 for 24 h at different concentrations or untreated. β‐tubulin was used as housekeeping gene. (B) Quantitative RT‐qPCR. Fold increase of c‐Myc and (C) PLK1 transcripts in the D17 cell line, after 12 and 24 h treatment with BI 2536 at different concentrations. Gene expression was normalized using the D17 untreated cell line; the error bars indicate the standard deviation of experimental triplicates

To investigate the effect of PLK1 inhibition on c‐Myc expression, WB and RT‐qPCR were performed on D17 cells exposed to BI 2536. As depicted in Figure 8 after 24 h of treatment with BI 2536, cells showed an evident concentration‐dependent decrease of c‐Myc protein expression. PLK1 protein showed a mild although non‐significant decrease when treated with BI 2536. Conversely, no significant variation in neither *PLK1* nor *MYC* gene expression was observed.

## 4 DISCUSSION

Transcription factors (TFs) are proteins regulating cellular gene expression that are mutated or dysregulated in a large number of canine and human cancer types.^34^, ^35^ TFs have been considered undruggable targets for a long time due to the lack of defined small‐molecule binding pockets.^36^ However, advances in the structural characterization of TFs and insights on their interaction with other proteins changed this postulate so far uncovering new therapeutic applications of TFs as cancer drug targets.^37^, ^38^

*MYC* is a member of a proto‐oncogenic TF family, which is deregulated in the majority of human and canine tumours, including hOSA. c‐Myc aberrant expression is associated with aggressive tumour behaviour and poor prognosis,^39^, ^40^ while its inactivation results in sustained tumour regression in murine models.^41^, ^42^ Nowadays, c‐Myc represents a long‐pursued target and a top‐listed putative target in anticancer therapy.^40^, ^43^ Among the multiple approaches blocking c‐Myc oncogenic activity, PLK1 inhibition by selective compounds, such as BI 2536 and BI 6727 (Volarsetib), represents an attractive therapeutic strategy in the treatment of c‐Myc‐driven tumours.^18^, ^19^ Indeed, both molecules induce c‐Myc protein degradation by inhibiting PLK1/Fbw7/c‐Myc axis via the suppression of Fbw7 auto poly‐ubiquitination.^44^

RNA sequencing analyses from a previous study showed an aberrant expression of both *MYC* and *PLK1* in two cOSA cell lines (D17 and D22) although no mutations were detected in WES analysis.^3^ Similarly, two recent studies reported an aberrant gene expression of *MYC* in cOSA samples.^45^, ^46^ In hOSA cell lines, c‐Myc overexpression is known to promote cell invasion via MAPK/ERK signalling.^16^ With this in mind, we aimed at investigating the role of PLK1 and c‐Myc in cOSA and evaluating the biological effects of BI 2536 in a well‐established cOSA cell line overexpressing both *PLK1* and *MYC* oncogene.

Since c‐Myc overexpression is known to predict outcome in hOSA,^12^, ^17^, ^47^ we hypothesized that expression of the PLK1 in addition to c‐Myc may replicate a similar result in cOSA. In this study, c‐Myc overexpression was associated to a negative outcome in our samples, supporting his role as an emerging prognostic biomarker.^48^ Conversely, although broadly expressed in cOSA samples, PLK1 did not correlate with the clinicopathological factors and c‐Myc expression. These data are in accordance with the human literature where PLK1 is an exploitable therapeutic target in c‐Myc overexpressing tumours^44^ but not a prognostic factor. This study also showed that dogs with grade III tumours had a significantly shorter DFI and OS compared to those bearing lower grade OSAs. This underlines the need for standardized histological criteria improving the controversial prognostic value of the grading systems applied to cOSA,^49^, ^50^, ^51^ as already achieved in hOSA with a 2‐tier grading system.^52^

To gain insight into the effects of PLK1 inhibition in cOSA cells, the *PLK1* and *MYC*‐overexpressing D17 cell line was treated with the BI 2536 molecule.^32^ Our results showed that PLK1 inhibition induced visible morphological changes after 24 h in a dosage‐dependent manner. Notably under 15 nM BI 2536 treatment, cells appeared rounded and detached from the plate; this biological effect has been previously described as indicative of mitotic arrest^53^ and was confirmed by our cell cycle analysis. Indeed, PLK1 inhibition induced a constitutive block of the D17 cell line in the G_2_/M phase. It is worth noting that, in untreated D17 cells, the peak corresponding to the G_2_/M phase of the cell cycle is likely due to superimposed polyploid cells in G_0_/G_1_. The increased third peak observed under BI 2536 treatment, likely represents the aforementioned polyploid cells blocked in G_2_/M, once again outlining the crucial role of PLK1 in the precise regulation of cell division.^54^ Consistently, BI 2536 treatment suppressed cell growth and induced mitotic arrest and cellular aneuploidy in the hOSA MG‐63 cell line.^22^ The potential antitumour activity of PLK1 inhibition was further corroborated in our study by the decreased cell proliferation and induction of apoptosis in treated tumour cells under matching in vitro conditions. The concentration‐dependent decrease in c‐Myc protein levels and the discrepancy with the transcript levels under BI 2536 treatment, highlighted the pivotal role of PLK1 inhibition on c‐Myc post‐transcriptional, proteasomal degradation and turnover.^18^ Surprisingly, PLK1 protein expression was not affected by the regulatory loop where c‐Myc activates PLK1 transcription in this study.^55^ Nevertheless, the complex interaction of signalling pathways regulating the cell cycle may have played a role in this discrepancy.^7^

A dosage‐dependent efficacy of PLK1 inhibition on cell cycle progression was observed after 16 h treatment which is the time required for cells to proceed through the mitotic process.^56^ Furthermore, the association between the constitutive block of cells in the G_2_/M phase with the evident decrease in c‐Myc protein levels underline the critical role of c‐Myc inactivation in cell cycle arrest coordinated by PLK1 inhibition,^57^ hence emphasizing the potential antiproliferative effect of PLK1 target inhibition in c‐Myc deregulated tumours.

This is a preliminary study investigating the expression of the PLK1‐c‐Myc signalling pathway and its potential role in cOSA. Nonetheless, the data obtained here are encouraging and show similarities to those retrieved in human cancer research, where PLK1 inhibitory molecules found an application in the treatment of several neoplasia^58^, ^59^, ^60^ promoting clinical trials with drugs indirectly targeting c‐Myc.^44^ Overall, our results highlight the effectiveness of PLK1 selective inhibition on c‐Myc and its downstream effects on cell proliferation and viability of cOSA cells. As previously described in hOSA,^19^ targeting the *MYC*‐driven signalling via the PLK1/Fbw7/c‐Myc axis might represent a promising therapeutic strategy for the treatment of canine patients bearing OSAs.

Although BI 2536 represents a successful proof of concept for successfully targeting this axis, future studies should include Volasertib in vitro assays. The promising preclinical efficacy and pharmacokinetic data of the latter led to its prioritization for clinical development.^20^ Therefore, the functional implications of Volasertib treatment and drug response on a number of cOSA cell lines need to be addressed to build an effective translational model. Finally, PLK1 also represents a central hub regulating functionality and interplay of several cell cycle checkpoints other than c‐Myc.^61^ Therefore, studies investigating the effects of PLK1 inhibition on the checkpoint activity the interplay with Fbw7 as regulator of the PLK1‐c‐Myc loop, and the interaction with other signalling pathways are critical to explore new combination therapies addressing the adverse effects and limitations of monotherapy.^62^

## CONFLICT OF INTEREST

None of the authors of this article has a financial or personal relationship with other people or organizations that could inappropriately influence or bias the content of the article.

## Supporting information


**Figure S1** Validation of immunohistochemistry antibodies using negative (canine subcutis and skeletal muscle) and positive (D17 cell line pellet) controls for PLK1; and negative (wall cell line pellet) and positive (D17 cell line pellet) controls for c‐Myc staining.

**Table S1** Immunohistochemical scoring
**Table S2** Primer sequences employed in RT‐qPCR

**Video S1** Supporting Information

## Data Availability

The data that support the findings of this study are available from the corresponding author upon reasonable request.
