# Supplementary figures and images for: The mitotic regulator polo‐like kinase 1 as a potential therapeutic target for c‐Myc‐overexpressing canine osteosarcomas
Source: Vet Comp Oncol. 2022 Aug 23;20(4):890–900. doi: 10.1111/vco.12854 (PMC9804590; doi:10.1111/vco.12854)

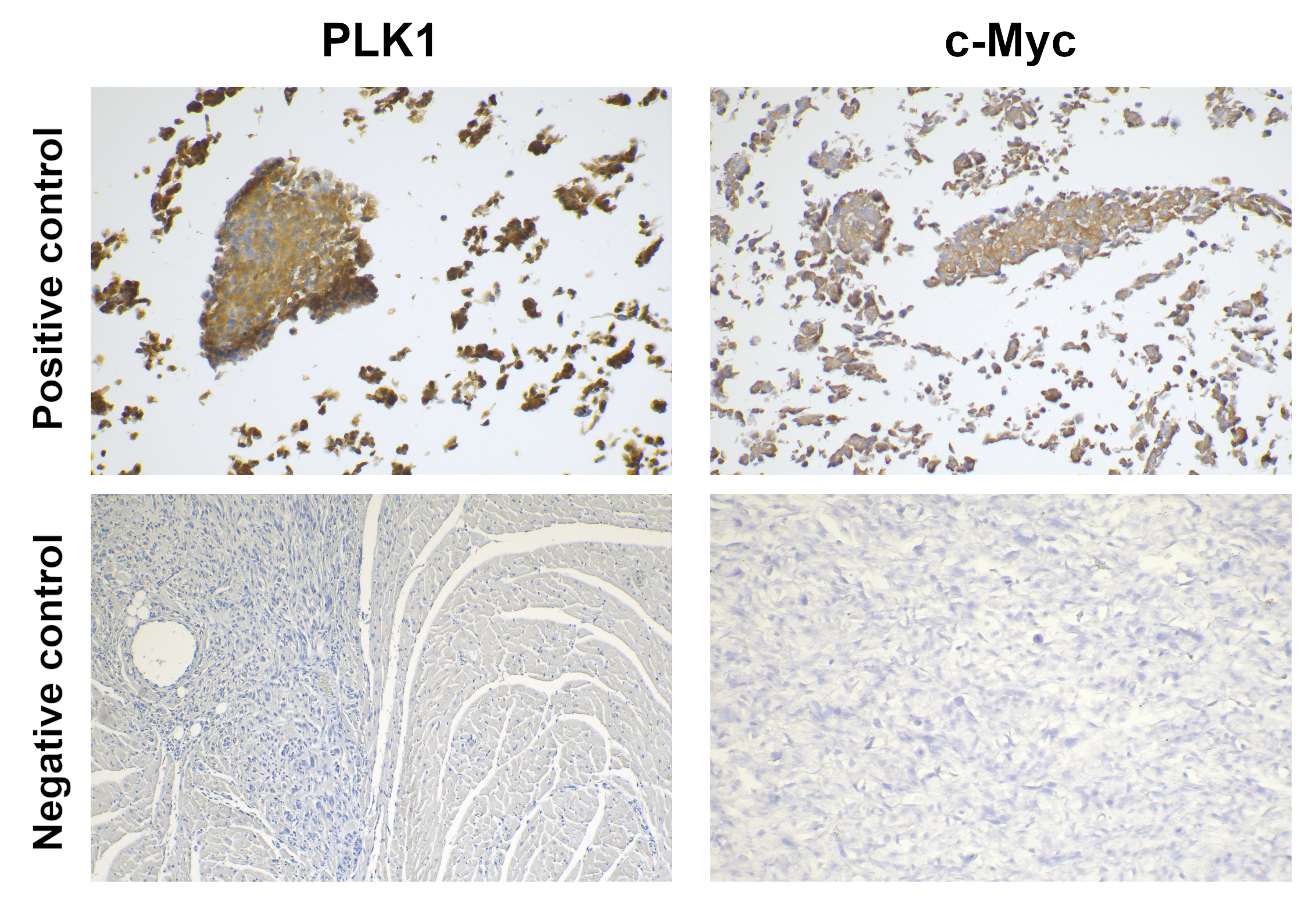

Supplement: Supplementary file 1 — Figure S1 Validation of immunohistochemistry antibodies using negative (canine subcutis and skeletal muscle) and positive (D17 cell line pellet) controls for PLK1; and negative (wall cell line pellet) and positive (D17 cell line pellet) controls for c‐Myc staining. [file VCO-20-890-s002.tif]
