# Supplementary material for: The mitotic regulator polo‐like kinase 1 as a potential therapeutic target for c‐Myc‐overexpressing canine osteosarcomas
Source: Vet Comp Oncol. 2022 Aug 23;20(4):890–900. doi: 10.1111/vco.12854 (PMC9804590; doi:10.1111/vco.12854)
Supplement: Supplementary file 2 — Table S1 Immunohistochemical scoring Table S2 Primer sequences employed in RT‐qPCR [file VCO-20-890-s001.docx]

Supplementary Table 1. Immunohistochemical scoring

| Antibody | % of positive cells | Immunoreactivity | Reference |
| --- | --- | --- | --- |
| c-Myc | 0 = negative 1 = 5-30%  2 = 31-50%  3 = 51-75%  4 = >75% | Low = 0-2  High = 3-4 | Fonseca-Alves et al. (2018) |

| Antibody | % of positive cells | Intensity | Immunoreactivity  [% x Int] | Reference |
| --- | --- | --- | --- | --- |
| PLK-1 | 0 = no staining  1 = <25%  2 = 25-50%  3 = 51-75% 4 = >75% | 0 = negative  1 = weak  2 = moderate  3= strong | Low = 0-7  High = 8-12 | Donizy et al.  (2016) |

Supplementary Table 2. Primer sequences employed in RT-qPCR

| Gene | Primer | Sequence |
| --- | --- | --- |
| PLK1 | Forward | 3’ GCATTGACGCTGTGTAGCTG 5’ |
|  | Reverse | 5’ AGCAACCGGAAGCCTCTTAC 3’ |
| c-Myc | Forward | 3’ CCCTCCACCAGGAAGGACTA 5’ |
|  | Reverse | 5’ CGTTGTGTGTTCGCCTCTTG 3’ |
| GAPDH | Forward | 3’ GGCACAGTCAAGGCTGAGAA 5’ |
|  | Reverse | 5’ CCAGCATCACCCCATTTGAT 3’ |
